# The Surgery of the Stomach

**Published:** 1914-06

**Authors:** 


**The Surgery of the Stomach.** By HERBERT J. PATERSON, M.A., M.C., M.B. Second Edition. Pp. xix, 342. London: James Nisbet & Co., Limited. 1914. Price, 15s. net.

This useful compendium of modern views of the surgery of the stomach has so quickly reached a second edition that but few words are needed in its praise.

New additions are stereoscopic photographs of the operation of gastro-enterostomy, which in our opinion are inferior to drawings in point of clearness. There are also some plates illustrating the use of X-rays after bismuth feeding, and the chapter on the physiology of gastro-enterostomy has been rewritten. The author holds that the value of gastro-enterostomy is due to its physiological rather than its mechanical effects. He refuses to accept the accumulated evidence of the development of cancer from ulcer, and would advise against the excision of ulcers of the stomach or an exclusion of the pylorus for duodenal ulcers.

